# Seeing the Whole Elephant: Imaging Flow Cytometry Reveals Extensive Morphological Diversity within *Blastocystis* Isolates

**DOI:** 10.1371/journal.pone.0143974

**Published:** 2015-11-30

**Authors:** John Anthony Yason, Kevin Shyong Wei Tan

**Affiliations:** Department of Microbiology, Yong Loo Lin School of Medicine, National University of Singapore, Singapore, Singapore; California State University Fullerton, UNITED STATES

## Abstract

*Blastocystis* is a common protist isolated in humans and many animals. The parasite is a species complex composed of 19 subtypes, 9 of which have been found in humans. There are biological and molecular differences between *Blastocystis* subtypes although microscopy alone is unable to distinguish between these subtypes. *Blastocystis* isolates also display various morphological forms. Several of these forms, however, have not been properly evaluated on whether or not these play significant functions in the organism's biology. In this study, we used imaging flow cytometry to analyze morphological features of *Blastocystis* isolates representing 3 subtypes (ST1, ST4 and ST7). We also employed fluorescence dyes to discover new cellular features. The profiles from each of the subtypes exhibit considerable differences with the others in terms of shape, size and granularity. We confirmed that the classical vacuolar form comprises the majority in all three subtypes. We have also evaluated other morphotypes on whether these represent distinct life stages in the parasite. Irregularly-shaped cells were identified but all of them were found to be dying cells in one isolate. Granular forms were present as a continuum in both viable and non-viable populations, with non-viable forms displaying higher granularity. By analyzing the images, rare morphotypes such as multinucleated cells could be easily observed and quantified. These cells had low granularity and lower DNA content. Small structures containing nucleic acid were also identified. We discuss the possible biological implications of these unusual forms.

## Introduction

*Blastocystis* spp. are protistan parasites found in humans and many types of animals. It is the most commonly isolated eukaryote in humans [1,2]. *Blastocystis* is a species complex comprising 19 subtypes (STs). ST1-9 have been found in humans and, with the exception of ST9, in other animal hosts as well. *Blastocystis* ST1 and ST3 are most frequently isolated, but there is geographical diversity in global distribution. For example, ST4 is common in Europe but not in the rest of the world [3]. ST2 and ST6 had a higher occurrence than ST3 in a study in Colombia [4] and ST4 has not been detected at all. This was also the case in a study in Argentina [5]. ST9 had the lowest occurrence and was only found in Denmark and Japan [3]. Subtypes of *Blastocystis* can be determined by genetic analyses, but STs also exhibit differences in size, morphology, growth in culture, host range, drug resistance, host immune response, adhesion to host cells and protease activities [6–10]. Studies have also indicated that ST identity may determine symptomatology and pathogenic potential [5,11]. While its pathogenicity is still yet to be elucidated, *Blastocystis* has been associated with gastrointestinal diseases [11]. It has also been implicated in waterborne disease outbreaks [12,13]. Due to its ubiquity and numerous animal hosts, it has the potential to be a threat in public health.

The conventional method for detection of *Blastocystis* is by microscopic observation in clinical (mainly fecal specimens) and environmental samples. This method, however, is not adequate to differentiate *Blastocystis* STs [6,14,15]. Under the microscope, *Blastocystis* appear round with a prominent central vacuole. The cytoplasm is usually located at the edges where the nucleus (or several nuclei) and other organelles can be found. Granular forms can also be observed and some multi-vacuolar forms have been reported [6,16]. Irregularly-shaped cells are also seen and these have been labelled as amoeboid forms in some studies [6,16,17].

There have been controversies on the life cycle of *Blastocystis* [18–21]. This is partly due to the unknown status of several morphological forms observed both in culture and clinical samples. For example, multivacuolar forms have been thought as *Blastocystis* undergoing multiple fission or schizogony similar to what happens in other protistan parasites [18]. There are also suggestions that amoeboid forms may undergo plasmotomy [22]. Whether or not granular forms have specific biological functions is still yet to be discovered [16]. There was also a report on granular amoeboid forms expelling small granules in xenic cultures [16]. The authors however did not investigate further if these granules represent reproductive stages. Currently, only binary fission has been accepted as the type of reproduction in *Blastocystis* since only this mode has been observed. Other investigators also suggest that other forms may be artefacts and do not represent reproductive stages [1,6,23]. There is, however, an interest on looking for alternative modes of reproduction. For some, binary fission alone could not explain the high number of cells in cultures and in fecal specimens [18]. Just like the parable of the blind men and the elephant, various groups have reported on distinct forms without taking into consideration the morphological complexity of the parasite. This often leads to biases and confusion in the *Blastocystis* field. There is therefore a need to evaluate morphological forms exhibited by *Blastocystis* in terms of their biological importance.

In this study, we used an imaging flow cytometer to survey the morphological characteristics of 3 *Blastocystis* subtypes (ST1, ST4 and ST7). The Amnis ImageStream Mark II is capable of acquiring thousands of images in a sample. These images can then be analyzed based on specific characteristics such as size, aspect ratio, fluorescence staining intensity, etc. We applied this technique to characterize morphological features such as shape, size, granularity and location of nuclei that can differentiate one subtype from the others. We also used fluorescence dyes to visualize cellular structures such as vacuoles and nuclei. We then calculated for the proportions of cells showing these features. This study is the first to provide a comprehensive and unbiased overview of the various morphological forms of *Blastocystis* in culture and sheds new light on the roles of certain forms of the parasite.

## Methods

### Parasite Axenic Cultivation

*Blastocystis* NUH9, WR1 and B isolates were maintained in 9ml Hyclone Iscove's Modified Dulbecco Medium (IMDM) (GE Healthcare Life Sciences, Logan, UT) supplemented with 10% horse serum (Life Technologies, Grand Island, NY). These isolates were previously axenized [24–26]. NUH9 was isolated during a health screening from an asymptomatic patient [26]; WR1 from Wistar rat [24]; and B from a patient complaining of diarrhea [25,27]. *Blastocystis* complete medium were pre-reduced for at least 12 hrs before use. Culture tubes were kept in in 5-L anaerobic jars with anaerogen gas pack (Oxoid, Basingtoke, UK) at 37°C.

### Ethics Statement

*Blastocystis* isolate B was obtained from an existing collection at the Department of Microbiology of the National University of Singapore (NUS). Human isolates were obtained from patients at the Singapore General Hospital in the early 1990s, before Institutional Review Board was established in NUS. NUH9 was isolated in 2007 from stool samples submitted for routine heath screening and approval from the National Healthcare Group Institutional Review Board was obtained before project commencement. All samples were anonymized.

### Subtyping of *Blastocystis* Isolates

The isolates were previously genotyped using primers based on the organism's small sub-unit ribosomal RNA (SSU rRNA) gene [26,28]. We also confirmed the isolates' subtypes using PCR protocol based on DNA from the parasite's mitochondria-like organelle (MLO) [29]. Total DNA were extracted from 1 x 10^6^ cells using Qiagen DNA stool kit (Qiagen, Hilden, Germany) following the manufacturer's instructions. PCR was perfomed using Q5 High-Fidelity 2X Master Mix (New England BioLabs, Ipswich, MA) with 500 ng of DNA sample. All PCR runs were completed using BioRad iQ5 thermocyler (Hercules, CA). PCR products were sequenced and compared to National Center for Biotechnology Information (NCBI) (USA) nucleotide sequence database to confirm subtype identities.

### Fluorescence Staining and Imaging Flow Cytometry

**Fig 1:**
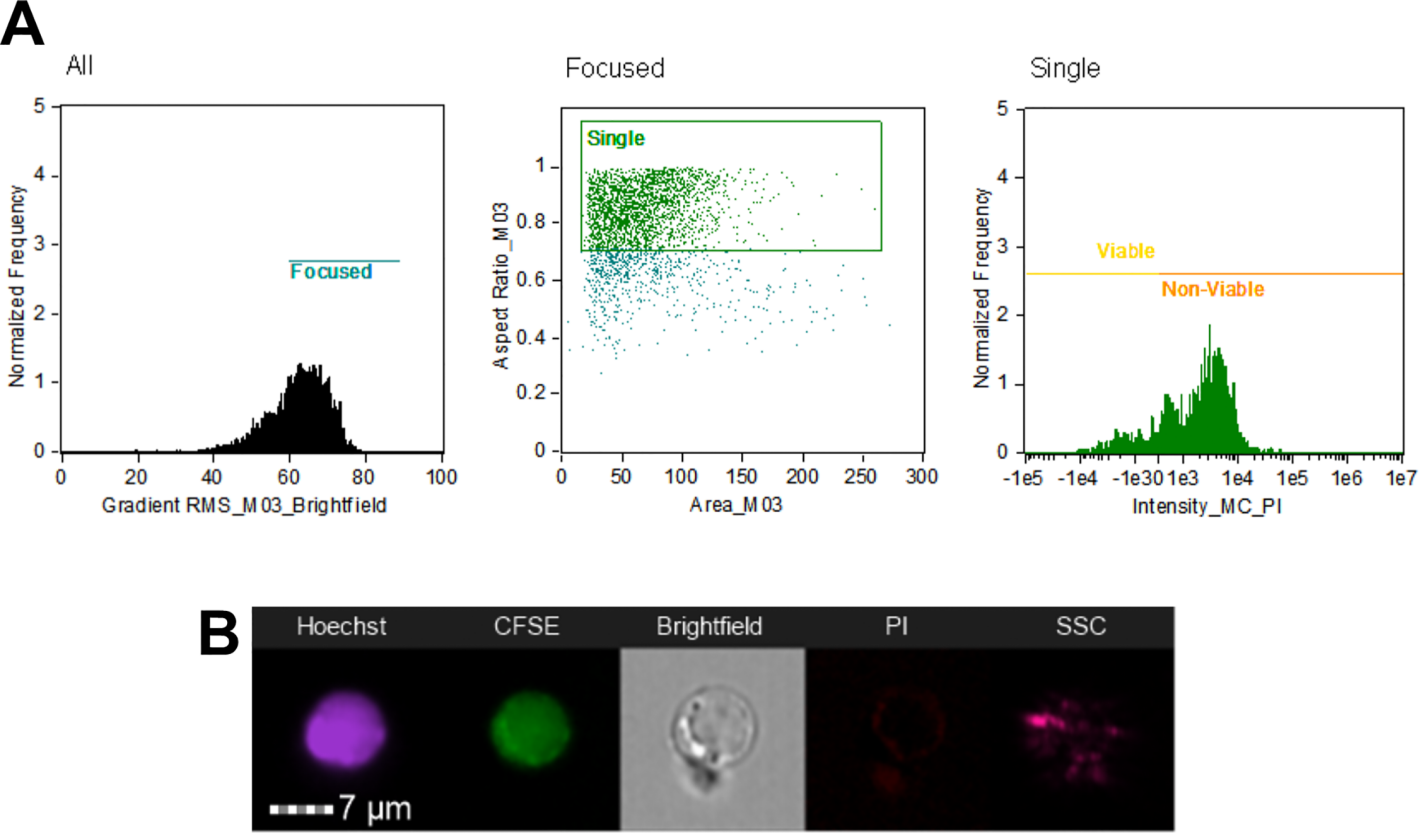
Initial gating strategy to analyze *Blastocystis* cells. Cells were gated for focused cells using brightfield channel, then selection of single cells using aspect ratio and area units, and finally to classify viable and non-viable cells using PI-staining characteristics. The above graphs shows the analysis for *Blastocystis* ST1-NUH9 isolate (A). Subsequent analyses made use of features arising from Hoechst and CFSE staining characteristics as well as features from brightfield and side-scatter channels (B).

In order to analyze cultures at the same stage of growth, 2-day old cultures of *Blastocystis* isolates WR1 and B and 7-day old culture of isolate NUH9 were harvested. The cells were washed twice by centrifugation at 1,000 x g using warm PBS. 2 x 10^7^ cells in 200 μL PBS were collected into 1.5 μL microtubes. The cell suspensions were then stained with 1 μg/mL propidium iodide (PI) (BioVision, Mountain View, CA), 5 μM carboxyfluorescein succinimidyl ester (CFSE) (Life Technologies, Eugene, OR) and 1 μg/mL Hoechst 33342 (Life Technologies, Eugene, OR) for 15 mins. PI stain was used to select for viable and non-viable cells. Cells will only take up PI when there is membrane disruption. CFSE is used in proliferation studies and is found to stain vacuolar compartments of *Blastocystis* [8]. Hoechst stains the DNA and is useful for cell-cycle analyses. Actively dividing cells will have higher emission while dying cells undergoing DNA fragmentation will have lower fluorescence readings. The cells were then washed to remove excess stains and fixed in 2% formaldehyde. Single stained cells were also prepared and used to create a compensation matrix. *Blastocystis* cell suspension heated to 80°C for 15 mins was used as positive control for PI-staining. We used Amnis ImageStream MarkII (Merck Millipore, Seattle, WA) with 4-laser attachment (375, 488, 561 and 642) to acquire *Blastocystis* cell images. 2,000 events were obtained with low flow speed at 60x magnification. Images at extended depth of field (EDF) setting were also acquired. EDF involves deconvolution to obtain highly focused images. Gating strategy involved selecting for focused cells using RMS gradient values, then for single cells using brightfield aspect ratio (Fig 1). Viable *Blastocystis* cells were identified as those without PI-staining. Cell shapes were characterized using aspect ratios from brightfield and CFSE staining. Acquisition was done using 3 different batches of cultures. Analysis of images was performed using IDEAS software version 6.1.

### Statistical Analyses

The proportion of populations based on shape and granularity were analysed for significant differences using Student’s two-tailed T-test. Hoechst-staining differences between groups based on circularity were analysed for significance using ANOVA. Statistical analyses were done using GraphPad Prism 5.0

## Results and Discussion

*Blastocystis* cultures of NUH9, WR1 and B isolates were harvested at the log phase of their corresponding growth curves. NUH9 has the slowest growth rate and cultures from this isolate were harvested after 7 days. Two-day old WR1 and B cultures were collected and used in the experiment. Subtyping of the isolates using MLO-based primers confirmed the identities of the isolates used: NUH9 is ST1, WR1 is ST4 and B is ST7. This step also made certain that each culture contain only one isolate/ST and was not contaminated by others.

**Fig 2:**
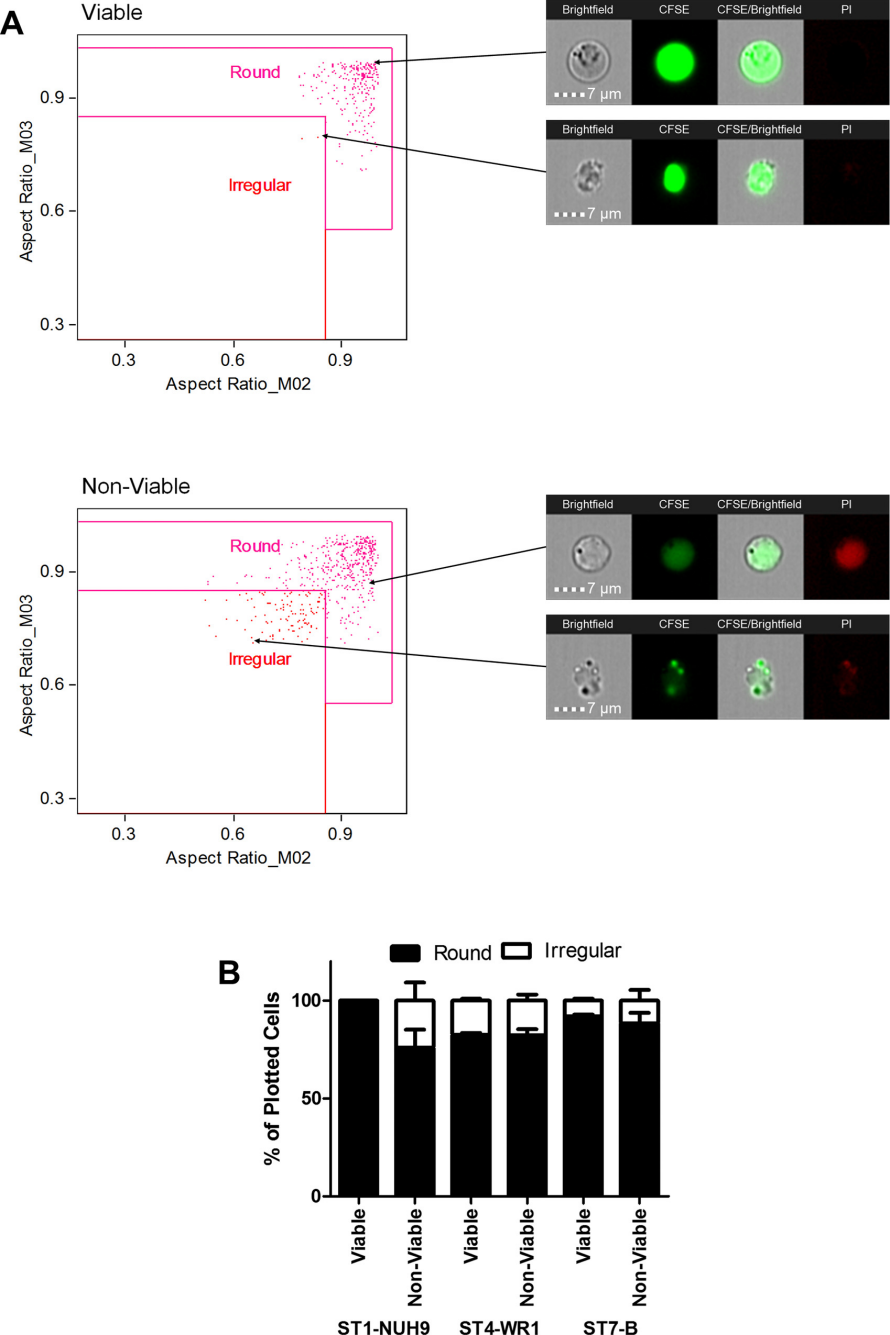
Gating for round and irregular shapes of *Blastocystis*. The shapes of *Blastocystis* were selected based on aspect ratios from the brightfield channel (M03) and CFSE staining (M02). Viable and non-viable cells were plotted separately. The above graphs shows the gating for *Blastocystis* ST1-NUH9 isolate (A). The average proportion of round and irregular shapes fond in cultures of *Blastocystis* ST1-NUH9, ST4-WR1 and ST7-B were then plotted in a graph (B). These are based on three separate batches of cultures and independent runs in ImageStream. Error bars signify standard error values. *p*-values comparing the proportion of round cells between viable and non-viable populations are 0.06, 0.47 and 0.31 for ST1-NUH9, ST4-WR1 and ST7-B isolates, respectively.

**Fig 3:**
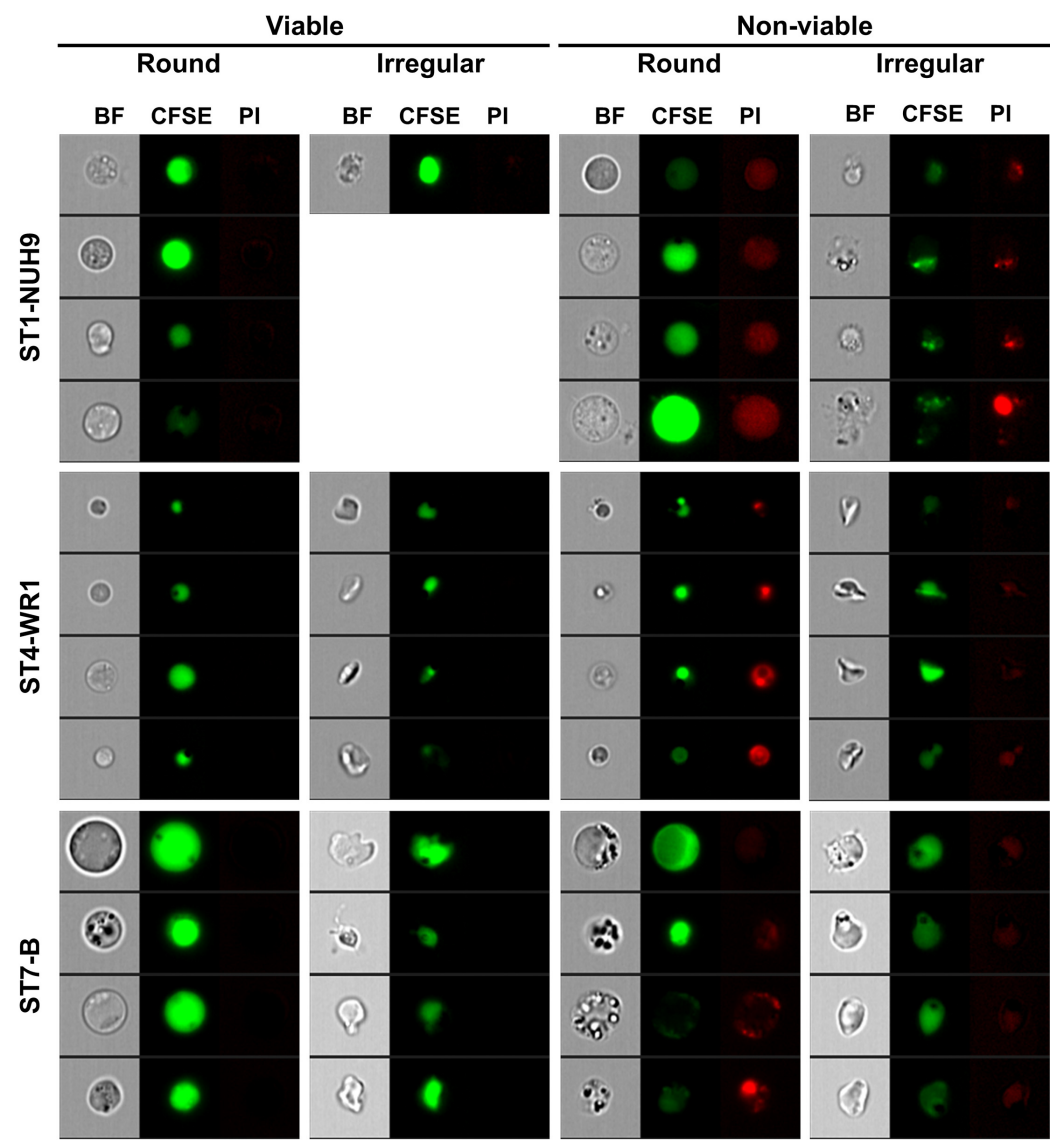
*Blastocystis* STs display various shapes from both viable and non-viable populations. Image gallery of *Blastocystis* cells showing round and irregular shapes from viable and non-viable populations. Each cell shown in the brightfield (BF) view has corresponding images which display CFSE and PI staining. The latter was used to determine viability. Irregular-shaped cells show elongated cells and amoeboid forms. Amoeboid forms with prominent pseudopodia and filamentous attachments are restricted to non-viable forms ST1-NUH9 and ST4-WR1. There are rare (0.1%) cells in viable ST7-B population that show an amoeboid-like morphology.

Using imaging flow cytometry, both round and irregular shapes were found in all *Blastocystis* isolates (Figs 2A and 3). The proportion of these shapes however differs from one subtype to another. Viable *Blastocystis* ST1-NUH9 is exclusively round, but only 76% round (the rest are irregular) in non-viable cells. Viable *Blastocystis* ST4-WR1 and ST7-B isolates are 83% and 92% round-shaped, respectively. These proportions are slightly lower (82% for ST4-WR1 and 88% for ST7-B) in non-viable cells (Fig 2B).

Several studies have attached significance to amoeboid forms in terms of reproduction and pathogenicity and these appear as irregularly-shaped cells [13,20]. These reports however did not identify the subtype of *Blastocystis* they have observed. Past studies also did not take into account the viability of the cells being observed. In this study, we were able to analyze morphological features of *Blastocystis* while determining their viability using PI-staining. We found that irregularly-shaped cells in ST1 are non-viable and therefore may not have any biological significance in this particular *Blastocystis* subtype. ST4 and ST7 isolates, on the other hand, have viable irregularly-shaped cells. This irregularity are due either to the cells' elongated shape or due to amoeboid features such as pseudopod-like structures (Fig 3). In our analyses, these amoeboid forms are rare among viable populations as most irregularly-shaped cells (especially in ST4) are elongated (Fig 3). In ST1 and ST4, these forms are more numerous but all are non-viable. Analysis of cells from viable ST7 isolates showed only one cell (out of ~1000 cells) that could be characterized as amoeboid (Fig 3). Experiments using different ages of cultures may be done in the future to determine the consistency of these rare cells in the population. Likewise, xenic cultures can also be investigated as suggested [30] noting that amoeboid forms may be more numerous in this type of culture condition.

**Fig 4:**
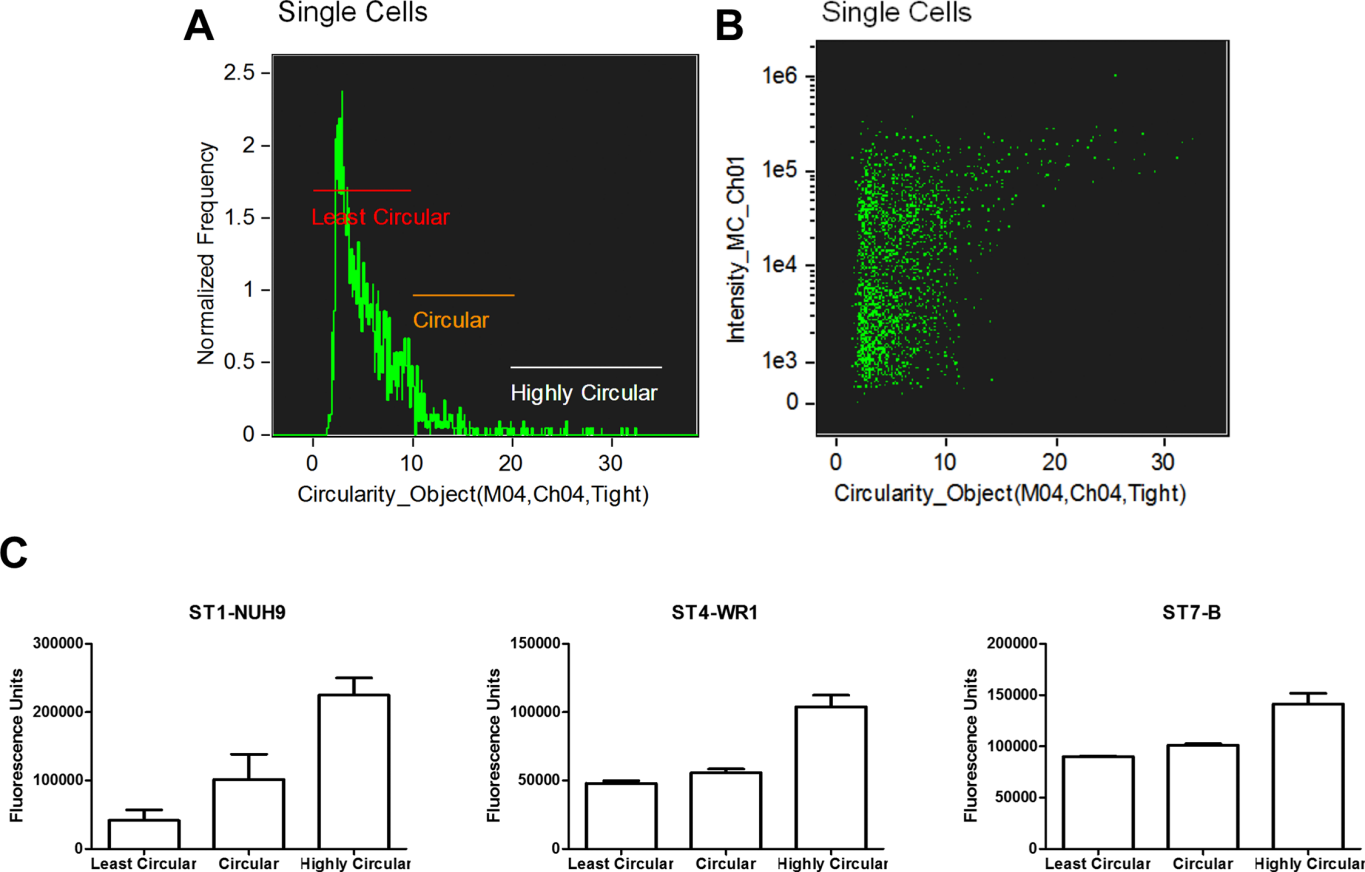
Round *Blastocystis* have higher DNA content. *Blastocystis* cells were plotted according to circularity using the software’s shape wizard. The population of cells was then divided into three groups: low, mid and high circularity (A). A dot-plot was generated to analyze the relationship between circularity and DNA content indicated by Hoechst staining of *Blastocystis* cells (B). The dot-plot shows that some highly circular cells have the highest DNA content while the least circular cells have the lowest DNA content. Bar graphs show the average Hoechst-staining of the three groups of *Blastocystis* cells based on circularity (C). Differences between groups based on circularity in each ST were found to be significant (*p* < 0.05) using ANOVA.

Analysis of single Hoechst-staining was also done to further correlate *Blastocystis* shape and reproductive status. Hoechst-staining is used for cell-cycle analysis. Actively dividing cells register higher fluorescence compared to inert cells. In all the isolates studied, round-shaped cells had higher average Hoechst-staining (Fig 4). This finding links well with the observation using PI-staining that most irregularly-shaped may not have biological or reproductive roles at all.

**Fig 5:**
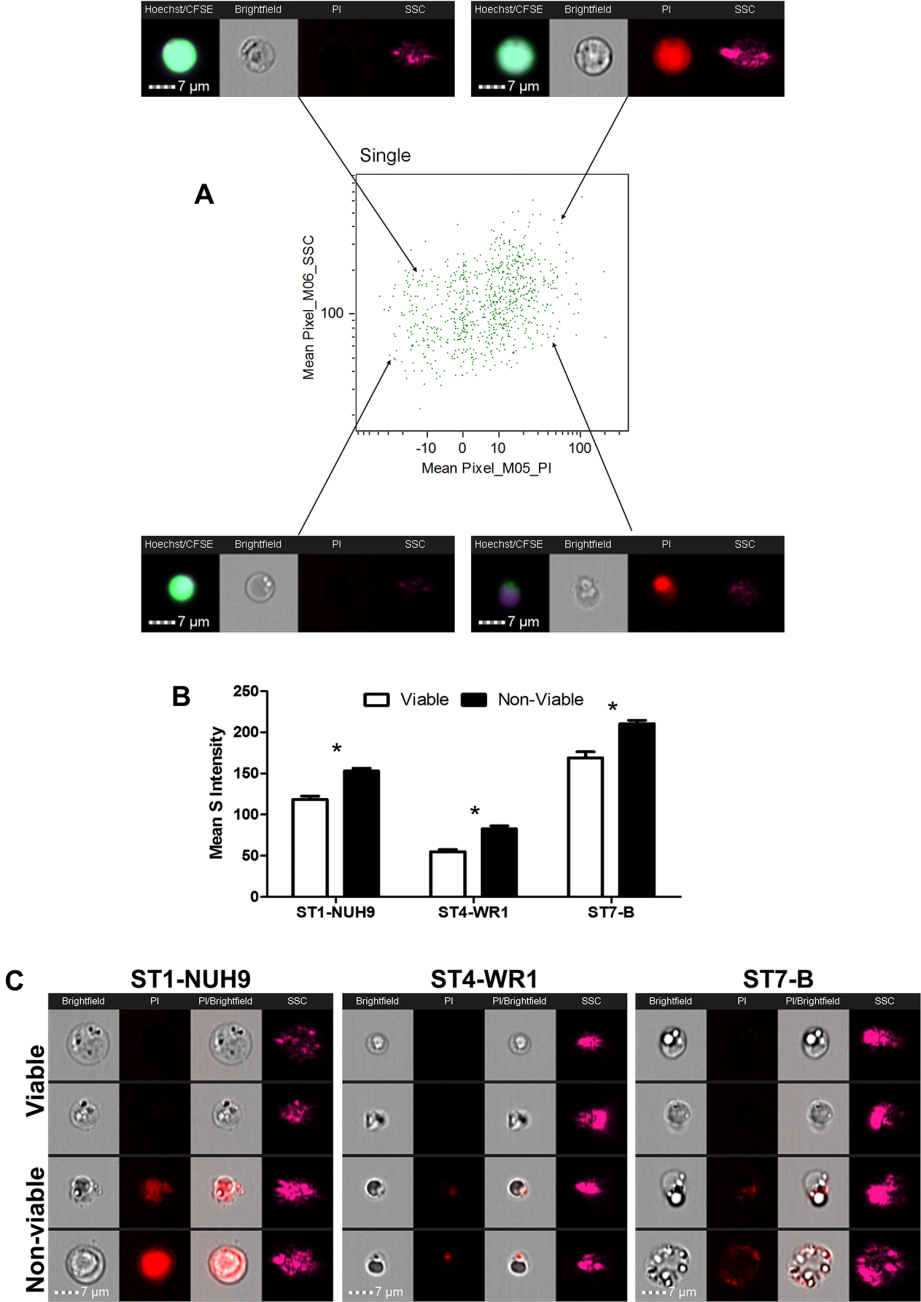
Analysis of *Blastocystis* based on granularity. *Blastocystis* populations were plotted according to mean pixel intensities of both side scatter channel and PI staining to determine the cells' granularity and viability, respectively (A). Cells with higher PI staining have more granularity compared to cells with lower PI staining. This observation is common to all subtypes used in this study as shown in a graph (B); *, *p* < 0.05. Sample images showing cells with high granularity among viable and non-viable populations (C). These cells may represent true granular cells and degenerating cells, respectively.

**Fig 6:**
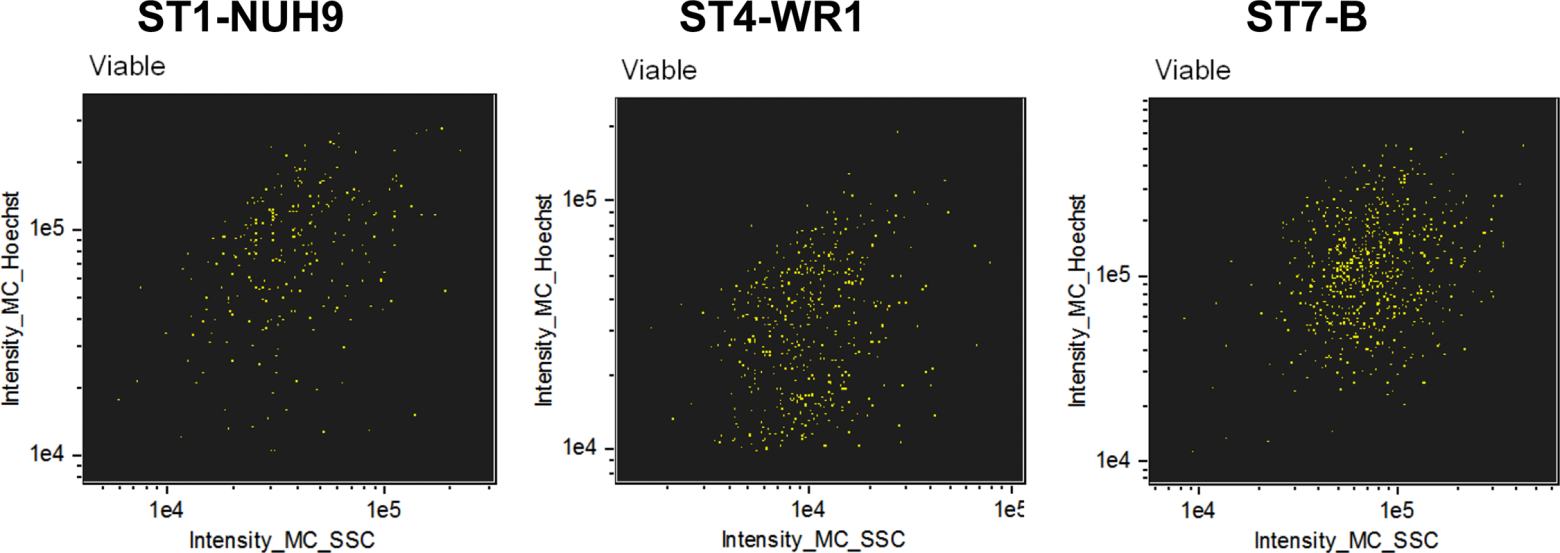
Viable *Blastocystis* cells with high granularity have higher DNA content. Dot-plot shows the populations of *Blastocystis* cells plotted according to circularity and Hoechst staining.

Aside from the round vacuolar forms, round granular forms are also commonly observed in clinical samples and in cultures [6,16]. Vdovenko [23] observed that these forms can naturally arise from vacuolar forms possibly by environmental exposure or fixation artefact. A more recent study has also mentioned this phenomenon [16]. In this study, we used the side-scatter channel to measure granularity of the cells. The average intensity coming from the channel in all STs in non-viable cells are higher compared to viable cells. Cells that are more granular can be found mostly among non-viable cells (Fig 5A). Non-viable cells have wider range in terms of granularity in contrast to viable cells where these tend to have lower side-scatter channel intensity. These findings are consistent among the 3 STs studied (Fig 5B). Nevertheless, cells that are more granular can also be found among viable cells. Our data suggests that there exists two populations of granular cells: true granular cells and those that are degenerating (Fig 5C) as reported previously [1,23]. We also add that granularity of these dying cells may be due more to environmental exposure rather than caused by fixation since we have observed the same proportion of granular forms in both fixed and unfixed samples (data not shown). We also used the Hoechst staining intensity of viable granular cells to find their biological significance. We have observe that these cells have higher DNA content compared to granular ones (Fig 6) These cells may therefore be in a more active reproductive state compared to less granular ones. We also analysed the granularity of multinucleated cells.

The diameter of *Blastocystis* vacuolar forms have been estimated to be between 2 and 200 μm [6]. A study MacPherson and MacQueen [31] indicated that the diameter of 80.8% of the *Blastocystis* they observed fell between 5 and 15 μm. This paper, however, was published before the genotyping era and so the particular ST of the isolate is unknown. In this study, we determined the size ranges of viable cells in each of the three subtypes. ST1-NUH9 and ST7-B isolates are bigger compared to ST4-WR1 isolates (Table 1). Furthermore, more than half (51.7%) of ST1-NUH9 cells have diameter of greater than or equal to 5 μm while only 37.3% of ST7-B cells have this size. ST4-WR1 cells are generally small with diameter of less than 5 μm in 98.3% of the population. Mirza et al. have previously reported differences in sizes between ST4 and ST7 isolates using flow cytometry [32]. In that study, the diameter of majority of *Blastocystis* cells after 48 hours of culture were 6 to 10 μm and 3 to 6 μm for ST7 and ST4 isolates, respectively. The calculations made for ST4 in this study mirrors that of the previous report. However, the measurement in this study for ST7 is smaller. The previous study has determined the sizes indirectly using known-sized beads and set them as standards for the forward scatter channel values in flow cytometry. This study however makes use of actual images of the cells and converting the pixel measurements to micrometers. We were also able to exclude doublets in measurement. There might be bigger-sized cells depending on the type and age of samples as well the amount of initial inoculum. Our analyses highlight the importance of taking into account the subtype used in any morphological studies. Our data suggest that the size range of *Blastocystis* is not as wide as between 2 and 200 μm, but narrower when ST identity is established. We also specifically measured viable cells only as the non-viable cells may feature degenerating cells and assume irregular shapes (Table 1).

**Table 1:** Viable *Blastocystis* size profiles indicated by cell diameter range and average diameter.

Subtype-Isolate	Diameter Range (μm)	Average Diameter (μm)	Cells with diameter ≥ 5 μm (%)
ST1-NUH9	2.7–8.7	5.0 ± 0.8	51.7 ± 6.1
ST4-WR1	2.2–6.2	3.6 ± 0.6	1.7 ± 0.4
ST7-B	3.2–7.5	4.9 ± 0.7	37.3 ± 4.5

**Fig 7:**
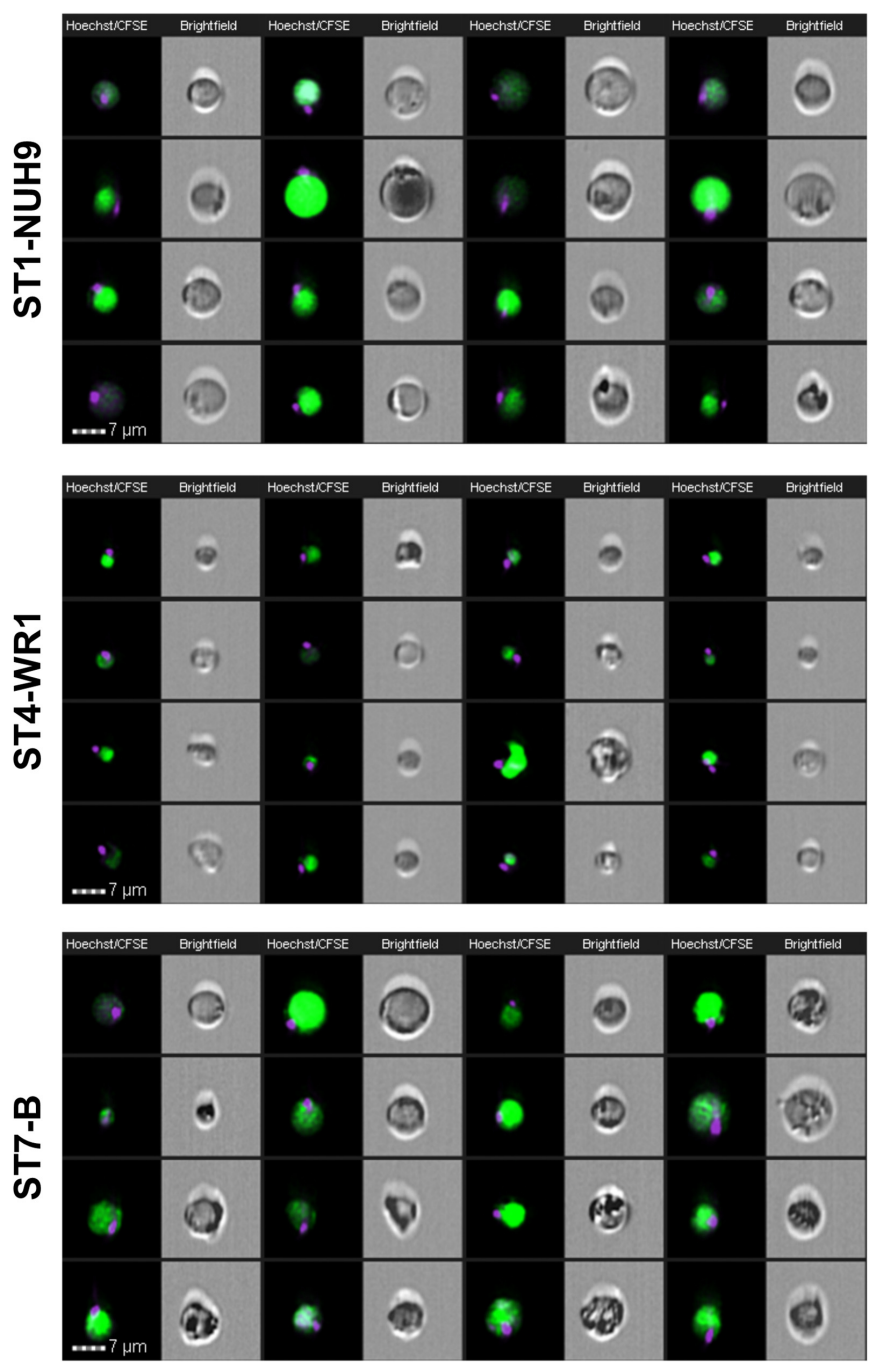
Image gallery of *Blastocystis* showing classical morphological forms at varying sizes in the viable populations. Images were acquired using EDF setting of the imaging flow cytometer. Each cell is represented by two images: Hoechst-CFSE-staining composite and brightfield image. The nuclei were stained with Hoechst and the vacuole with CFSE. These round forms show a single nuclei located at the edge of the cell. These forms comprise more than half of the population in all the subtypes studied.

**Fig 8:**
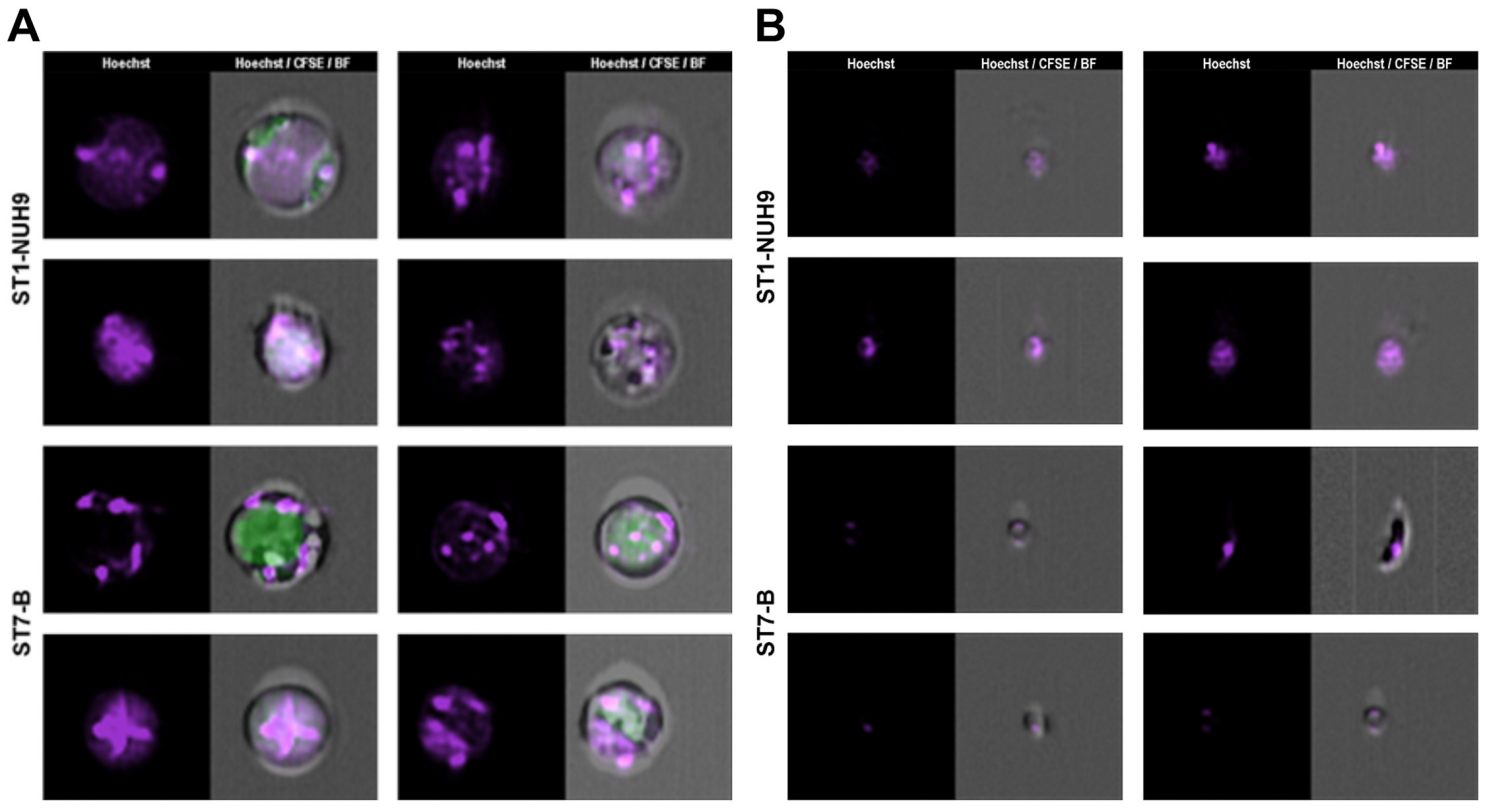
Image gallery of rare *Blastocystis* multinucleated cells. The nuclei is visualized by Hoechst staining. The nuclei number more than three and some appear to be concentrated in the center and not along the edges (A). Some of these cells also appear to show nuclear condensation. (B) Image gallery showing small structures which contains DNA.

**Fig 9:**
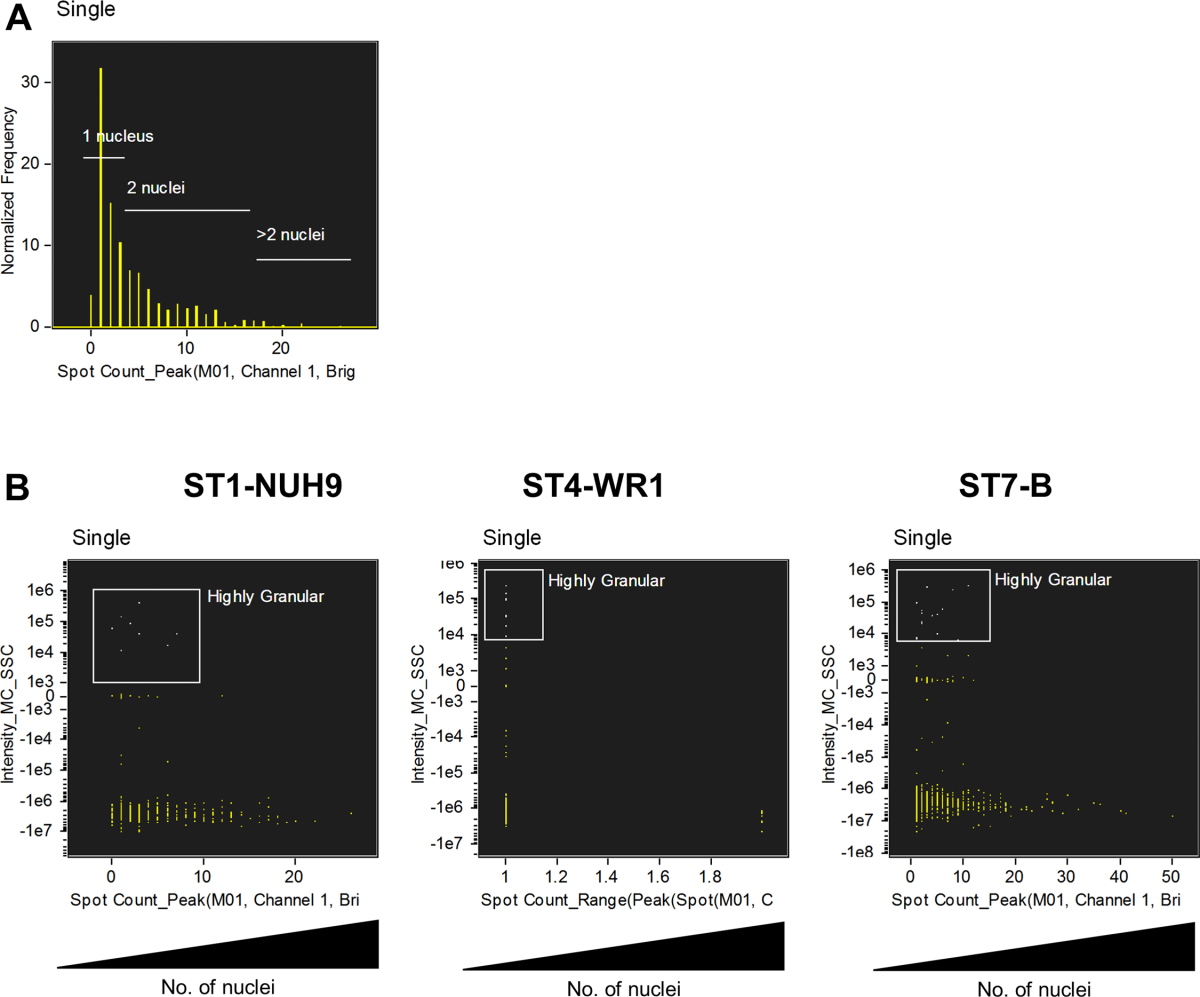
Multinucleated *Blastocystis* cells do not display higher granularity compared to uninucleated cells. The spot wizard of the IDEAS software was used to group cells based on number of nuclei. This was done by counting the ‘spots’ stained with Hoechst. The images were then visually analysed to determine the precise spot values that correspond to the number of nuclei (A). Dot-plots were generated for each ST to analyse granularity in multinucleated cells. These plots show that cells with high granularity are found in the populations of uninucleated cells.

We used Hoechst staining and the EDF setting of the imaging flow cytometer to analyze nuclear arrangement of viable *Blastocystis* cells. More than 80% of the population from the three subtypes studied featured 1–2 nuclei (Fig 7) located at the edge of the cells. ST4-WR1 isolates all feature one or two nuclei (and not more). This reflects the previous observation [31] whereby 98.4% of the cells have a similar arrangement. This study therefore supports the classical representation of *Blastocystis*. More infrequently, in the other STs (1–2% in ST1 and 2–8% in ST7), other nuclear features were observed. These cells either show more than 2 nuclei or that the nucleic acid stain covers a large area of the cell (Fig 8). The latter show an apparent nucleic acid condensation which may represent cells about to undergo binary fission. Surprisingly, we have observed multinucleation only in round cells in these two subtypes. We did not find evidence to support alternative modes of reproduction such as plasmotomy and budding in *Blastocystis* as suggested by others [16,18,22] since we did not observe multinucleation in irregularly-shaped viable cells. Some of the multinucleated cells show the nuclei located in the center of the cell with the size of the central vacuole diminished (as indicated by lower intensity of CFSE staining) (Fig 8A). If this is a reproductive stage, it may be an evidence of a multiple fission event in *Blastocystis* as suggested [33]. These, however, showed lower granularity (Fig 9). Cells with single or two nuclei also showed higher DNA content. This is consistent with the data above (Fig 6) and these suggests that multinucleated cells may not be in active reproductive stage as compared to the other cells. We have also observed small structures containing nucleic acid (Fig 8B). These structures are rare (2–4% of viable cells). They also do not appear to have vacuoles as indicated by low CFSE staining but consist mainly of nucleic acid material in its compartment. Our experiments did not determine whether these structures originate from bigger and multinucleated cells. It may also be possible that these represent starving cells or a state where *Blastocystis* sheds off its vacuole.

## Conclusions

In this study, we analyzed three STs of *Blastocystis* based on shape, size, granularity and nuclear arrangement using high content imaging flow cytometry. These features may be used to compare one *Blastocystis* ST to another. We have also identified rare multi-nuclear forms which are unlikely to represent an alternative mode of reproduction in the parasite. It would be interesting to investigate other isolates coming from the same or other STs so that inter- and intra-subtype variations in morphology can be determined. We used axenic cultures of *Blastocystis* but it would also be attractive to investigate cells from xenic cultures or from fecal samples. The methods outlined in this study can also be used to detect changes in *Blastocystis* upon treatment of drugs or modification of culture conditions. Data from this study provides an important and useful reference on the morphological profile of *Blastocystis* STs.

## Supporting Information

S1 Table Summary of Proportions of viable *Blastocystis* morphological forms and range of granularity found in each population of the three STs studied. (DOCX)
