# Supplementary material for: Seeing the Whole Elephant: Imaging Flow Cytometry Reveals Extensive Morphological Diversity within Blastocystis Isolates
Source: PLoS One. 2015 Nov 30;10(11):e0143974. doi: 10.1371/journal.pone.0143974 (PMC4664278; doi:10.1371/journal.pone.0143974)
Supplement: S1 Table — (DOCX) [file pone.0143974.s001.docx]

S1 Table. Summary of Proportions of viable *Blastocystis* morphological forms and range of granularity found in each population of the three STs studied.^a^

|  | ST1-NUH9 | ST4-WR1 | ST7-B |
| --- | --- | --- | --- |
| Shape^b^ |  |  |  |
| Round (%) | 99.3±0.2 | 82.5±1.6 | 92.0±1.6 |
| Irregular (%) | 0.7±0.2 | 17.5±1.6 | 8.0±1.6 |
|  |  |  |  |
| No. of nuclei^c^ |  |  |  |
| 1 (%) | 47.9±28 | 78.9±11.1 | 61.1±17.3 |
| 2 (%) | 50.3±27.8 | 21.1±10.6 | 34.2±19.6 |
| Others^d^ (%) | 1.8±0.2 | 0.0 | 4.7±2.9 |
|  |  |  |  |
| Small structures with Hoechst staining (%) | 2.1±0.1 | 0.0 | 4.4±3.3 |
|  |  |  |  |
| Size |  |  |  |
| Cells with more than or equal to 5 um diameter (%) | 51.7±6.1 | 1.7±0.4 | 37.3±4.5 |
|  |  |  |  |
| Granularity^e^ |  |  |  |
| Minimum (mean pixel units) | 22.4 | 15.4 | 19.8 |
| Maximum (mean pixel units) | 504.8 | 245.3 | 667.1 |

^a^calculated from three runs of experiments with each run having at least 2,000 events

^b^based on aspect ratios of brightfield image and CFSE staining of viable cells

^c^determined by analysis of Hoechst staining of EDF-enhanced images

^d^includes cells with more than 2 nuclei and cells that show outsized Hoechst staining

^e^determined from side-scatter channel of ImageStream
